# Some Near- and Far-Environmental Effects on Human Health and Disease with a Focus on the Cardiovascular System

**DOI:** 10.3390/ijerph17093083

**Published:** 2020-04-29

**Authors:** Germaine Cornelissen Guillaume, Denis Gubin, Larry A Beaty, Kuniaki Otsuka

**Affiliations:** 1 Halberg Chronobiology Center, University of Minnesota, Minneapolis, MN 55455, USA; 2 Department of Biology, Medical University, Tyumen 625023, Russia; 3 Executive Medical Center, Totsuka Royal Clinic, Tokyo Women’s Medical University, Tokyo 169-0071, Japan

**Keywords:** co-periodisms, heart rate variability, melatonin, physiopathology, stroke, weather

## Abstract

Environmental effects on human physiopathology are revisited herein from a chronobiologic viewpoint, with a focus on the cardiovascular system. Physiological variables undergo recurring changes that are predictable in a statistical, albeit not deterministic way. Biological rhythms cover a broad range of frequencies, which are usually shared by the environment as “co-periodisms”. Some of these photic and non-photic periodicities shared by the environment and physiopathology are reviewed herein, together with their possible underlying mechanisms. A plausible cascade of events from the long-period cycles found in the cosmic environment to those affecting the Earth’s atmosphere and weather conditions is presented, which may shed light on how they may shape the cycles characterizing human health. Maps of important cycles shared between the environment and physiopathology are being catalogued in an atlas of chronomes with the goal of distinguishing between strong and weak associations and providing an estimate of the lag that can be anticipated before observing physiological changes.

## 1. Introduction

The environment profoundly affects health in a variety of ways, either directly by exposing people to harmful agents or indirectly by disrupting life-sustaining ecosystems [1]. While widespread poverty and severe lack of public infrastructure (access to drinking water, sanitation, and lack of health care) are environmental hazards affecting countries in the early stages of development, environmental risks in wealthier countries primarily stem from urban air and water pollution [1]. The public health care burden of cardiorespiratory diseases caused by chemical air pollutants (CO, O_3_, SO_2_, CS_2_, NO_2_) and particulate matter has escalated during the past few decades in developing countries throughout the world. The high occurrence of acute and chronic obstructive respiratory disorders, lung cancer, and cardiovascular morbidity and mortality are linked to the adverse effects of air pollution and cigarette smoking [2]. Air pollution can trigger heart attacks, strokes, and irregular heart rhythms, particularly in people already at risk for these conditions [3,4]. Inhaled fine particulate matter reaches deep into the respiratory system, causing systemic inflammation, bronchitis, and pneumonia [4]. As reviewed in [4], the increase in systemic inflammation can predispose to endothelial dysfunction and platelet aggregation with an increase in C-reactive proteins, leading to atherosclerosis, myocardial infarction, carcinogenesis, and osteoporosis. Air pollution also decreases heart rate variability, which is a risk factor for cardiovascular diseases [4]. The potential molecular mechanisms of particulate matter-related cardiovascular diseases include direct toxicity to the cardiovascular system or indirect injury by inducing systemic inflammation and oxidative stress in the circulation [4,5].

There is, however, another aspect to the intimate link between environment and health, in which chronobiology and chronomics play a central role. Most, if not all, physiological functions are neither constant nor randomly varying, but undergo partly predictable recurring changes. Likewise, the incidence of various disease conditions undergoes partly predictable rhythmic patterns. Biological rhythms are critically important and were viewed by the late chronobiologist Franz Halberg as “the essence of life”. They cover a wide range of frequencies, usually shared by the environment as co-periodisms since, like all life forms throughout evolution, humans live in an open environment [6]. Among them are the circadian rhythms, which have gained great interest in light of their ubiquity, endogenous nature (now firmly documented at the molecular level), and wide-ranged involvement in health and disease [7]. Circadian rhythms are orchestrated by the suprachiasmatic nuclei as the brain’s primary pacemaker [8]. Clock genes are present in almost every cell. Circadian disruption can play a role in a wide range of pathology such as the increased risk for cardiometabolic disease and malignancy among shift workers. Likewise, neurodegeneration can cause circadian dysfunction, suggesting a bidirectional relationship between circadian rhythms and human health [9]. Single nucleotide polymorphisms in key circadian clock genes identified by genotyping large human populations have also been associated with disease conditions such as metabolic syndrome and high blood pressure. Moreover, shift work, which disrupts circadian rhythms, was reported to increase the risk of cardiovascular disease and cardiovascular disease mortality in a graded–response way [10].

Herein, some environmental effects are revisited, with emphasis on the cardiovascular system. Some near-environmental effects are related to Earth’s weather, as processes in the atmosphere may influence it, while other far-environmental effects are related to space weather. Based on periodicities shared between the broad environment and physiopathology, a possible cascade of events is presented that could account in part for some of the long periodicities found in a number of variables pertaining to human health.

## 2. Ubiquitous, Partly Endogenous Circadian Rhythms Synchronized by Light

Circadian rhythms, with a period of about 24 h, are partly endogenous, as first demonstrated by free-running: they persist under constant environmental conditions, albeit assuming a period slightly, but statistically significantly, different from exactly 24 h [11]. Circadian rhythms are primarily orchestrated by a small brain area of roughly 20,000 (in rodents; 200,000 in humans) neurons situated in the hypothalamus, directly above the optic chiasm, known as the suprachiasmatic nuclei (SCN). The SCN receive input from specialized photosensitive ganglion cells in the retina via the retinohypothalamic tract. Neurons in the ventrolateral SCN have the ability for light-induced gene expression. Melanopsin-containing ganglion cells in the retina have a direct connection to the ventrolateral SCN via the retinohypothalamic tract, where the signal allows synchronization of an organism’s circadian rhythms to the environmental light–dark cycle [12]. Thus, there is a clear exogenous influence by the environment on endogenous circadian rhythms. 

The onset of myocardial infarction and death from myocardial infarction are circadian periodic [13]. Most epidemiologic studies show a morning peak in onset. Overall, the incidence of myocardial infarctions is 19% higher-than-average around 09:45 and 16% lower-than-average around 18:00 [14]. A morning peak also characterizes the incidence of strokes (52% more likely to occur around 10:00 and 46% less likely to occur around 02:45), sudden death (34% more likely around 09:45 and 42% less likely around 02:45), and other cardiovascular diseases (25% more likely around 08:15 and 18% less likely around 12:15) [14]. Circadian patterns of adverse vascular events have been associated with circadian rhythms that modulate the hemostatic system including a morning increase in platelet aggregability, concentrations of coagulant factors and blood vessel viscosity, and the concomitant decrease in fibrinolytic activity; blood pressure and heart rate are also higher during the active part of the day and lower during rest/sleep [15]. The incidence of paroxysmal supraventricular tachycardia, of broadly classified ventricular arrhythmia, and of atrial fibrillation is also circadian periodic [16].

## 3. Effects of Weather on Human Physiopathology

While light is the primary synchronizer of circadian rhythms, ambient temperature and the day length are factors underlying the manifestation of circannual rhythms, notably at higher latitudes. Morbidity and mortality from respiratory and cardiovascular disease peak during the coldest months of the year in both the Northern and Southern Hemispheres. Their large-amplitude circannual rhythm follows the well-recognized and undisputed seasonal pattern affecting human health [17]. A larger incidence during the cold season also characterizes heart failure in both hemispheres [18] and ischemic strokes and intracerebral hemorrhages in Finland [19]. The prominence of the circannual rhythm in overall and cardiovascular mortality depends on latitude, as shown in data compiled from 19 countries, amounting to over 54 million deaths [20]. Several risk factors underlying adverse cardiovascular events such as body mass index, blood pressure, low-density lipoprotein (LDL) cholesterol, triglycerides, and glucose concentrations also follow a circannual rhythm [21]. A circannual variation has also been reported in plasma viscosity, fibrinogen, and factor VII activity with elevated concentrations in the cold season, potentially generating a “hypercoagulable state” that could account for a rise in cardiovascular morbidity and mortality [22]. Plasma viscosity, red blood cell deformability, whole blood viscosity, hemoglobin, hematocrit, mean corpuscular volume, platelet count, α1-glycoprotein, fibrinogen, plasminogen activator inhibitor-1, LDL cholesterol, and triglyceride concentrations, measured longitudinally in a few healthy volunteers, also assumed maximal values during the cold season, when high-density lipoprotein cholesterol and cortisol reached their minimum [22].

Circannual-dependent effects of air pollution on acute mortality were studied in Wuhan, China [23]. Statistically significant interactions were reported between three pollutants and cause-specific mortality. The strongest effects occurred consistently during the cold season for all-natural, cardiovascular, stroke, and respiratory mortality. The study showed a clear seasonal pattern of acute mortality effects of ambient air pollution, the strongest effects occurring during the cold season [23]. Seasonal changes in vitamin D have also been implicated as a factor contributing to the circannual variation in cardiovascular deaths [24]. Ambient temperature, however, is the dominant factor underlying the higher incidence of mortality in the coldest months. In a study of daily temperature and mortality from cardiovascular disease and myocardial infarction from nine Chinese mega-cities during 2007–2013, statistically significant nonlinear associations between temperature and mortality were observed, with a total of 195,516 cardiovascular events and 50,658 deaths from myocardial infarction attributable to non-optimal temperatures [25]. The impacts of atmospheric temperature and pressure on daily rates of myocardial infarction and coronary deaths were studied as part of the MONICA (acronym for “monitoring trends and determinants in cardiovascular disease”) project [26]. Associations between an increase in coronary heart disease occurrence and low atmospheric temperatures have been reported from mortality data and hospital admission registries. In this study of 257,000 men, 25 to 64 years of age, 3616 adverse events occurred during the 10 years from 1985 to 1994. The rate of events decreased linearly with increasing atmospheric temperature: a 10 °C decrease was associated with a 13% increase in event rates. A V-shaped relationship with atmospheric pressure was detected with a minimum of daily event rates at 1016 mbar. A 10-mbar decrease below 1016 mbar and a 10-mbar increase above 1016 mbar were associated with a 12% and 11% increase in event rates, respectively [26]. Effects of temperature and atmospheric pressure were found to be independent, influencing both coronary morbidity and mortality rates, with stronger effects in older age groups and for recurrent events [26].

## 4. Effects of Atmospheric Temperature and Pressure on Stroke Events

Statistics for 2014 in Minnesota [27] indicate that about 2.2% adults reported ever having had a stroke in their lifetime, affecting over 90,000 people. Over 5% (2172) of all deaths are due to stroke. The cost for the over 12,000 hospitalizations for acute stroke events is estimated at $418 million. Compared to Caucasians, African-Americans have a 25%, and Asians have a 30% higher stroke death rate [27].

**Figure 1:**
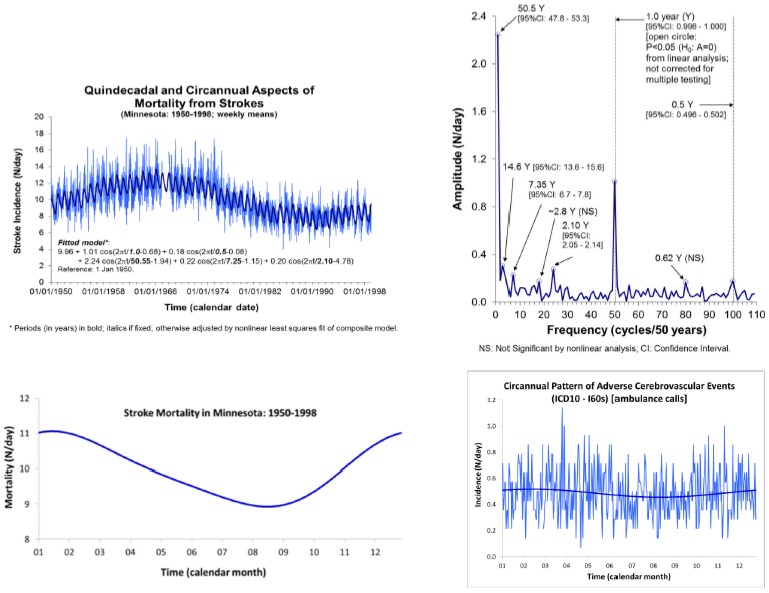
Time course of the incidence of stroke mortality in Minnesota during the span from 1950 to 1998, shown with 4-component model including a prominent circannual rhythm (top left). Least squares spectrum illustrates how prominent the circannual variation is, and indicates the presence of a weak second harmonic with a period of 6 months (top right). Reconstructed waveform of the circannual rhythm shows a peak incidence in winter (bottom left). Similar results are found for the incidence of all adverse cerebrovascular events in Khanty, Siberia, during the span from 2001 to 2014 (*N* = 2485), based on much fewer data collected from calls for an ambulance (bottom right). © Halberg Chronobiology Center.

**Figure 2:**
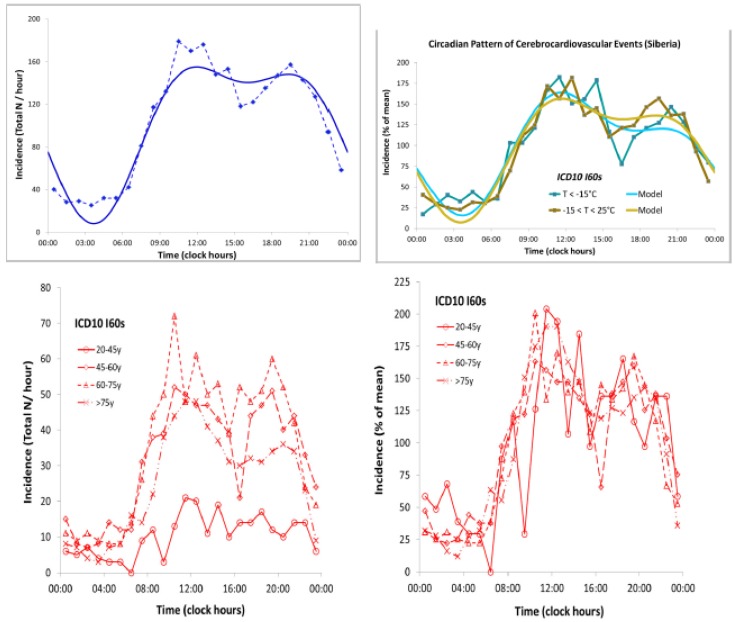
Circadian rhythm in the incidence of all adverse cerebrovascular events in Khanty, Siberia, during the span from 2001 to 2014 (*N* = 2485) shows the morning increase with a secondary smaller peak in the evening (top left). A similar pattern is found at all age groups (bottom), notably after the data are expressed as a percentage of mean (bottom right). A similar circadian pattern is also seen irrespective of environmental temperature (top right). © Halberg Chronobiology Center.

Our analysis of the daily incidence of strokes in Minnesota during the span from 1950 to 1998 showed the presence of about-yearly cycles peaking in the winter, an about weekly variation, a half-year component characterizing the waveform of the circannual variation, and other periodicities that had counterparts in our broader environment (Figure 1) [28]. A similar yearly pattern characterizes the incidence of all adverse cerebrovascular events in Khanty, Siberia, according to ambulance calls’ records (Figure 1). In Khanty, a morning peak characterizes the circadian variation, as expected, which is similar in different age groups (Figure 2). A model consisting of cosine curves with periods of 24 and 12 h approximates the circadian variation well, irrespective of atmospheric temperature (averaging either less than 15 °C or 15 to 25 °C), when data are expressed as a percentage of the daily mean value (Figure 2).

The influence of weather (temperature, humidity, and barometric pressure) on the incidence of stroke was investigated in Yokohama, Japan, based on computerized records of ambulance transport between January 1992 and December 2003 (N = 53,585 cases; 30,163 men, 23,421 women, one unknown) [29]. The study targeted patients aged 50 years or older who were transported by ambulance and coded as stroke patients (ICD-9:430–438; ICD-10:I60–I67) and identified according to the first diagnosis by doctors in the emergency department. The influence of daily meteorological conditions on the daily incidence of emergency transport events coded as stroke was examined by Poisson regression analysis, also accounting for the occurrence of any holiday including Sunday and all national holidays. Meteorological conditions were divided into 96 weather pattern categories, and the incidence of stroke events per 100,000 per day was calculated in relation to the 96 patterns. Both temperature and relative humidity were found to be negatively related to the incidence of stroke events in both men and women. The lowest incidence was reported on Sunday, and the highest on Monday. High-risk weather was also found to be associated with a larger morning peak in the circadian pattern, characterized by a secondary smaller peak in the evening [29]. A systematic review and meta-analysis of the effect of ambient temperature on stroke occurrence concluded that lower mean ambient temperature was significantly associated with the risk of intracerebral hemorrhage, but not with ischemic stroke and subarachnoid hemorrhage, and that larger temperature changes were associated with higher stroke rates in the elderly [30].

The circaseptan pattern of lower incidence on Sunday and higher incidence on Monday of stroke events in the Yokohama study [29] was in agreement with our results from different studies. Similar results were found in relation to (1) the 98,625 stroke events recorded in relation to ambulance calls made in Moscow during 1979 to 1981 (*p* < 0.001) [14]; (2) those of daily stroke incidence in Minnesota during 1968 to 1996 (*p* = 0.032) [28]; and (3) events in Khanty on days when atmospheric temperature was above 25 °C (*p* = 0.026). Circaseptan as well as circadian and circannual patterns of stroke incidence may vary, however, depending on their etiology, as shown in a study in Lund, Sweden on a relatively small number of 497 events between September 1987 and August 1988 [31]. For instance, the incidence of subarachnoidal hemorrhage peaked later in the day compared to large vessel disease infarction or infarct due to cardiac embolism [31].

## 5. Periodicities in Weather Conditions

It is well known that atmospheric temperature undergoes marked changes along the scales of the day and the year, at least at latitudes away from the equatorial zone. Less well known, however, are environmental counterparts for the about-weekly patterns in the incidence of strokes and other cardiovascular conditions, albeit any causal relationship will need further investigation. An about-weekly periodicity in rainfall has been reported by Abbot [32]. We detected a near- but not exactly 7-day component in the global geomagnetic disturbance index Kp [33], a finding later extended by Vladimirskii et al. [34] to the antipodal geomagnetic index aa, which goes back to 1868. A circaseptan pattern with a period of about 6.74 days was also detected in data on local geomagnetic pulsations from a stand-alone magnetometer in the relatively less polluted Antarctic, 600 km from the nearest human habitation [35]. In the Antarctic, the amplitude of the local natural component was larger than that of an also present second anthropogenic precise 7-day component. Interestingly, circaseptans were already known to Hippocrates, Galen, and Avicenna, who observed their manifestation in the onset of an illness [36,37]. Circaseptans were also the topic of a book on periodic diseases [38].

Temperature and rainfall are also characterized by an about 33- to 35-year periodicity, the so-called BEL cycle, named after Brückner, Egeson, and Lockyer [39]. Charles Egeson can be credited with the first publication on a cycle in weather said to be of 33 years length, documented by charts of time series covering 110 years. Within a few months, extensive documentation of transtridecadal changes was provided by Brückner. Lockyer associated this transtridecadal component with the period he found to characterize changes in the duration of the about 11-year solar activity cycle [39]. Our analysis of Brückner’s data taken off his published figure estimated the period of temperature to be 36.8 (95% CI: 32.6, 41.0) years and that of rainfall to be 33.6 (95% CI: 29.9, 37.4) years [40]. Similar results apply to other variables recorded by Brückner: 40.6 (95% CI: 37.4, 43.9) years for the frequency of cold winters; 35.8 (95% CI: 31.4, 40.2) years for the duration of ice-free rivers; and 36.3 (95% CI: 33.3, 39.3) years for wine harvest [39]. Analysis of heliogeomagnetic data available from the OMNIweb database identified proton temperature, sigma (Bx), plasma speed, and Kp as potential counterparts, with periods of 34.3 (95% CI: 27.0, 41.6), 31.9 (95% CI: 24.7, 39.0), 33.0 (95% CI: 20.1, 46.0), and 32.7 (95% CI: 28.3, 37.0) years, respectively [41].

## 6. Effects of Space Weather on Human Physiopathology

There is merit of longer-term forecasts of both meteorological conditions and space weather. Mapping long-term periodicities can play an important role in this respect. Time series analysis can also help explain how climatic changes may be affected by space weather. Non-photic cycles related to space weather have periods, among others, of about a week, about 0.42 year (solar flares), about 1.3 years (solar wind speed), and about 11, 22, and 35 years (sunspots). As reviewed below, all have documented counterparts in human physiology and pathology, albeit shared periodicities do not necessarily imply the existence of a causal relation. 

Assessing influences of space weather on human physiology and pathology often relies on correlations between the socio-biological variable and the environment. One serious pitfall of such an approach is the disregard for periodicities characterizing both biology and inanimate nature, many of them shared between the two systems. Correlations notoriously lead to spurious results in the presence of rhythms, as the correlation coefficient is largely dependent on the phase difference of a given periodic component between the two variables. Indeed, if two variables share the same periodicity and are in phase, the correlation will be positive, but if they are opposite in phase, the correlation will be negative, and if they are in quadrature, the correlation will be near zero. In the presence of cycles, methods other than the Pearson product-moment correlation are advocated. Cross-spectra and coherence spectra are one option, as are superposed epoch analysis and the remove-and-replace approach [42].

An influence from solar activity has been known for a long time. Data reported in 1922 already showed an increase in symptoms of patients on days with versus days without sunspots [43]. Whether considering all or only severe symptoms (such as those related to the heart and vessels, liver, kidney, and nervous system), their incidence is higher on days with sunspots (*p* < 0.001). Using more rigorous methods than the Pearson product-moment correlation, some of the periodicities shared by the environment and biology are briefly reviewed, with a focus on the cardiovascular system.

### 6.1. Circaseptans

The very young, the elderly, and otherwise vulnerable individuals are thought to be particularly affected by space weather. The nonlinearly estimated period of the prominent about-weekly variation of blood pressure and heart rate of newborns [35,44], synchronized by the time of birth (development) rather than by the day of the week (social influence), correlates with the circaseptan period of the local geomagnetic activity index obtained during matching spans [45]. Adults also display a more prominent about-weekly variation in heart rate when a circaseptan component characterizes the rate of change in sunspot areas, as detected using a remove-and-replace approach [46]. In both cases, results were modulated by an about 10.5-year component similar to the solar activity cycle [42,46]. Similarly, a circaseptan component characterizes mortality from myocardial infarction, as shown in the Republic of Georgia during the span from 1980 to 1982 when solar activity was high and the interplanetary magnetic field underwent an about-weekly variation, but not during the span from 1984 to 1987 when solar activity was down, as were circaseptans in the interplanetary field [47].

### 6.2. Circatrigintans

The Sun, composed of a gaseous plasma, rotates with different periods at different latitudes. Julius Bartels’ Number is the serial count that numbers the apparent rotations of the Sun as viewed from Earth, used to track certain recurring or shifting patterns of solar activity. In this system, each rotation has a length of exactly 27 days, close to the synodic Carrington rotation rate, starting on 8 February 1832 as day one. 

Traute and Bernhard Düll collected and evaluated meteorological data and thousands of death certificates between 1 January 1928 and 31 December 1932; they stacked the mortality data over 68 consecutive 27-day solar rotation cycles along the scale of Bartels’ Numbers [48]. They could thus align patterns in the daily incidence of mortality from different causes with those of relative sunspot numbers and worldwide magnetic characters, assessed separately for each day of the 27-day Bartels’ cycle after stacking. They documented clear 27-day patterns for all-cause mortality as well as for death attributed to nervous and sensory system diseases, suicides, diseases of blood circulation, and from respiratory diseases [48]. Compared to peak mortality from cardiovascular diseases, deaths attributed to nervous and sensory system diseases peaked earlier and deaths from respiratory diseases peaked later. Possible differential effects of space and/or Earth weather were thus shown with respect to suicide and other deaths associated with the nervous and sensory systems versus death from cardiac or respiratory disease as well as overall death by differences in the phase of a common 27-day cycle characterizing these mortality patterns [48]. 

### 6.3. Cis-Half-Year

The cis-half-year with a period of about 0.42 year, which characterizes solar flares [49], is also modulated by an about 10.5-year cycle, as found in a 40-year record of around-the-clock self-measurements of heart rate by a clinically healthy man [50]. In these data, a spectral analysis revealed the presence of prominent yearly and half-yearly components in addition to a less prominent about 0.42-year component. Fitting this 3-component model to data in a 4-year interval progressively displaced by a 0.2-year increment, the 0.42-year component is detected with statistical significance only when the total solar flares index is high. Its amplitude follows an about 10.5-year pattern lagging that of solar flares [50]. A cis-half-year also modulates circulating melatonin, sampled around the clock serially-independently from each of 172 clinically healthy individuals examined between October 1992 and December 1995 [51]. It is further a prominent spectral peak in transverse data on uric acid [52] and in the incidence of sudden cardiac deaths in several geographic locations [53].

### 6.4. Transyear

Beyond a cis-half-year, uric acid, and the incidence of sudden cardiac deaths are also characterized by a transyear, with a period of about 1.3 years, detected in data on solar wind speed [54]. A transyear is present in all 43 longitudinal records of blood pressure and heart rate available for analysis thus far [53,55]. It also characterizes the daily incidence of mortality from myocardial infarction in Minnesota during the span from 1968 to 1996 [28] and the incidence of suicides in Minnesota during the span from 1968 to 2002 [56]. In addition to solar wind speed, cosmic rays (in Chicago), solar magnetism, Wolf numbers, coronal index, Kp, aa, and Dst also showed an about 1.3-year periodicity [53,55]. 

### 6.5. Multidecadals

**Figure 3:**
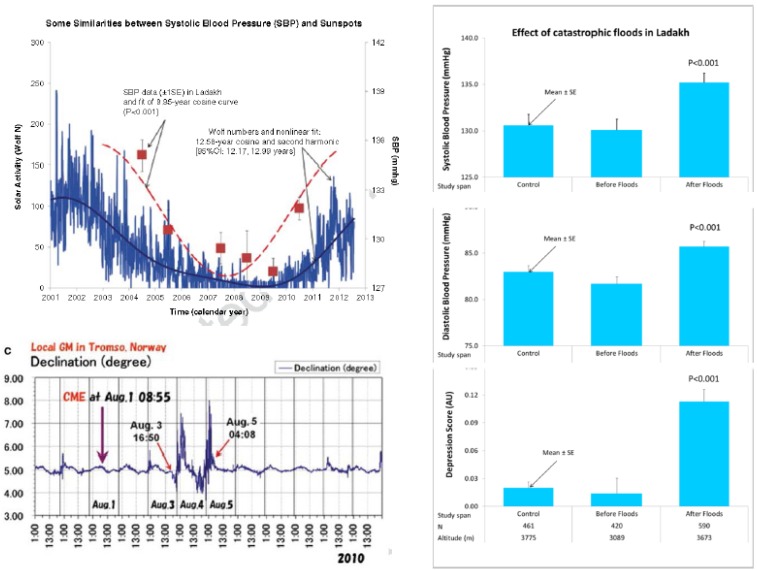
Systolic blood pressure follows an about 11-year cycle similar to that of solar activity in Ladakh (top left). A strong magnetic storm was followed by catastrophic floods in Ladakh (bottom left), where studies monitored the cardiovascular health of populations living at different altitudes. This event was associated with drastic changes in blood pressure and mood (right) [60]. © Halberg Chronobiology Center.

The about 10.5-year component characterizing mortality from myocardial infarction in Minnesota during the span from 1968 to 1996 accounts for 5% excess deaths during years of maximal solar activity compared to years of minimal solar activity [28]. Circadecadal cycles have been documented in individual records of physiological variables (blood pressure, heart rate, and heart rate variability), in bacterial sectoring, and organismic resistance (gauged by a 15-year record of urinary excretion of 17-ketosteroids by a clinically healthy man) [57,58]. These numerical near-matches of biota with the solar activity cycle, organized as a sequence of events (gauged by the relative phase relations of biological extrema following extrema in solar activity) are only hints, but additional results from spectral coherence and superposed epoch analysis suggest an association with natural physical environmental factors for several aspects of human morphology, physiology, and pathology. A histogram summarizing the best-fitting periods (determined by nonlinear least squares) in the low-frequency spectral region for all longitudinal records available to us for analysis showed a clear peak around 10.5 years [6,59]. In Ladakh, where studies monitored the cardiovascular health of populations living at different altitudes, systolic blood pressure was found to follow an about 10.5-year cycle similar to that of solar activity in the population as a whole [60]. There, a strong magnetic storm was followed by catastrophic floods. This event, in turn, was associated with drastic changes in blood pressure and mood (Figure 3).

## 7. Possible Underlying Mechanisms

Evidence has been presented for the effect of space weather on human pathophysiology, reflected in several shared periodicities, but the question remains regarding what the precise mechanism(s) may be. Just as solar events have a profound effect on electric and electronic networks, they may equally affect electrical systems in the heart and brain.

Heart rate variability is a candidate mechanism underlying the influence of space weather on myocardial infarction [28]. Not only is heart rate variability decreased in patients after a myocardial infarction, a lower heart rate variability is also predictive of future development of coronary artery disease. Furthermore, heart rate variability is lower in cosmonauts on the MIR station monitored during a magnetic storm when compared to those monitored during quiet conditions [61]. Similar results were obtained in 7-day/24-h electrocardiographic records from clinically healthy individuals in Alta, Norway, located above the Arctic Circle [62], where a graded response of heart rate variability was associated with the severity of geomagnetic activity [63]. Heart rate variability was decreased primarily in the low-frequency range of the spectrum that may relate to variations in the activity of the renin–angiotensin system and thermoregulation and to the baroreceptor modulation of the sympathetic and vagal nervous tone, but not in the high-frequency spectral range thought to reflect the respiratory modulation of vagal nerve activity [64].

Suppression of melatonin secreted by the pineal gland, possibly via desynchronized biological rhythms, is another plausible mechanism underlying space weather effects on physiopathology [65]. Suppression of the nocturnal melatonin metabolite 6-hydroxymelatonin sulfate was reported after exposure to 50/60 Hz magnetic fields as well as in association with increased geomagnetic activity [66,67]. Disruptions in the circadian rhythmicity of pineal melatonin secretion have indeed been associated with certain depressive disorders in humans [68], conditions thought to respond to magnetic fields [69]. Although most humans are not consciously aware of the geomagnetic stimuli encountered in everyday life, two classes of ecologically-relevant rotations of Earth-strength magnetic fields can produce strong, specific, and repeatable effects on human brainwave activity in the EEG alpha band (8-3 Hz), suggesting the presence of a ferromagnetic transduction element such as biologically-precipitated crystals of magnetite (Fe_3_O_4_) [70]. Other mechanisms have also been considered: calcium ions in cells could play a role in one or more mechanisms [65,71]; Schumann resonance signals could be the global environmental signal absorbed by the human body, thereby linking geomagnetic activity and human health [65]. Such a mechanism could account for the observation that daily autonomic nervous system activity not only responds to changes in solar and geomagnetic activity, but is synchronized with the time-varying magnetic fields associated with geomagnetic field-line resonances and Schumann resonances [72,73,74]. Stochastic resonance has also been invoked [75]. It is unlikely, however, that a single mechanism can explain all of the reported phenomena.

Schumann resonances are a set of spectral peaks in the extremely low frequency portion of the Earth’s electromagnetic field spectrum. They are global electromagnetic resonances, generated and excited by lightning discharges in the cavity formed by the Earth’s surface and the ionosphere, where important chemical reactions may be impacted by space weather with consequences on Earth in terms of cloudiness and rainfall. Interestingly, some of the non-photic cycles characterizing space weather such as the about 1.3-year transyear, are observed in phenomena taking place in the ionosphere. This is the case, for instance, in concentrations of hydrogen peroxide and higher organic peroxides, which undergo variations in the transyear range in addition to a prominent circannual variation, as seen in data measured hourly between 1998 and 2005 at the Zugspitze/Hohenpeissenberg Station, Platform Hohenpeissenberg (47°48′ N, 11°01′ E, elevation: 985 m above mean sea level) [76]. It is thus possible that non-photic components in biology may respond not only to changes in geomagnetic disturbances linked more directly to space weather, but also to other factors from the near environment such as the meteorological variables. 

If so, a trickle-down effect of the prominent solar activity cycle, which modulates non-photic cycles such as the transyears and cis-half-years, may have repercussions on Earth, mediated by phenomena occurring in the Earth’s atmosphere, notably the ionosphere, the ionized part of the Earth’s upper atmosphere. The ionosphere plays an important role in atmospheric electricity as it forms the inner edge of the magnetosphere. Cycles similar to the solar activity cycle have been detected in data on the total cultivated area and total production of wheat and rye, barley, corn, soya, and sunflower in Romania, where, on average, all five crops followed an about 10.7-year component during the span from 1968 to 2000 [77], perhaps mediated by meteorological conditions. It should be remembered that an about 11-year cycle was first reported in economics by Hyde Clarke [78]. Changes in solar activity, which have also been associated with changes in the weather by others [79], are thought to influence economic cycles [80]. As an example, merchandise exported to India (1708–1734) follow about 11-year cycles [81,82,83]. 

It would not be surprising then, that such partly predictable changes in weather, agriculture, and economics more broadly, may have a potential bearing on the health of pregnant women, their newborn babies at the time of birth, and early development [84]. Barker’s “developmental” model for the origins of a wide range of chronic diseases links their cause to variations in fetoplacental development, thought to lead to variations in the supply of nutrients to the baby that permanently alter gene expression [84]. In a study of demographic data in historical Norway, the lifespans of individuals born during a span of solar maximum were found to be 5.2 years shorter than those born during a span of solar minimum, while fertility and lifetime reproductive success were reduced among low-status women [85]. Anthropometric measures at birth have also been found to follow about 10.5- and about 21-year cycles [57], perhaps in response to cycles in agriculture, following cycles in weather and climate. Both components characterize land air and sea surface temperature in North America, a global temperature index [86]. Global temperature data are also available for the Southern Hemisphere. Increasing global temperature poses several threats including loss of land, possibly irreversible adaptation of phytoplankton to long-term high CO_2_, with consequences on the entire food chain. 

## 8. Conclusions

This review highlighted the effects of atmospheric and space weather on human health. While the exact nature and mechanisms of action still need to be better understood, some adverse effects of space weather have been related to the occurrence of magnetic storms. Triggered by solar emissions, it takes about one to three days for the effects to be felt on Earth, thus leaving enough time for a warning to be issued. Such a warning system is already in place such as those used in aviation and other human endeavors. Applications in the healthcare system have also emerged.

Research is ongoing to develop rooms that compensate for external magnetic fields [87], and magnetically-shielded wards are already available in a Moscow hospital to care for cardiovascular patients [88]. Discussions are underway to add information on space weather to improve on the forecast now provided based on meteorological conditions. While these endeavors focus on short-term predictions, there is also a need for longer-term predictions to optimize the management of available resources locally, and perhaps also globally. 

Precisely because non-photic environmental cycles are notoriously wobbly, mapping their non-stationary characteristics in specific frequency ranges as they change over time may lay the foundation to research which features of space weather may influence what aspects of human pathophysiology. For this purpose, at the Halberg Chronobiology Center, an atlas of chronomes (broad time structures, defined by their rhythm characteristics: period, amplitude and phase) is being prepared to better understand broad environmental influences on human health [89,90]. It will include maps of important cycles shared between the environment and physiopathology. Matching period length and comparing phases at common periods will distinguish between strong and weak associations and provide an estimate of the lag that can be anticipated before observing physiological changes. The atlas will organize charts in a way that conveys sequences of events in order to facilitate making connections between the presence of non-photic cycles in the cosmos, how they may be affecting the ionosphere and influence weather on Earth, and how these changes can impact agriculture, nutrition, the presence of pathogens, and overall human health.

## Dedication

This manuscript is dedicated to the memory of Paul Rosch, with whom the authors shared many of the scientific interests briefly reviewed herein.
